# Supplementary material for: Chromothripsis during telomere crisis is independent of NHEJ, and consistent with a replicative origin
Source: Genome Res. 2019 May;29(5):737–49. doi: 10.1101/gr.240705.118 (PMC6499312; doi:10.1101/gr.240705.118)
Supplement: Supplemental Material [file supp_gr.240705.118_Supplemental_file_1.zip › contigs/annotated_contigs/DB81/contig.2.DB81_length_401_mean_cov_6.89775561097.docx]

**DB81_length_401_mean_cov_6.89775561097**

```
ACCAAAAAAACAAAAAAAAAGAGTCACTTCAACAGCAGTGAGATCACCTTAAATAAAATGCTGGGCCTGTAGATACCACCTCAGCCAGG
 >chr8:106508597-106508902 - E=4e-168
AGATTAAGTTCAACCTCACCAGAATAAGATCTATGAACATTGTCTGCTTCCTGACATGATGCACTGGGAAAGGCATATTATTTTTGTTT

TATTCTTGCCCCAAACACATAACTTCAATCTAATCATGATAAAATATTAGAAACAAACCCAAATTTAGAGACAGTCTACTAGATATTTG

AGTACCACTCTCCAAAAGTGTCTTGAAAGACAGGGAAGA|CTGAG|TATATATATATACTTTTGGTGGCAGCTTAAATGTGAGCTAAAG
                                             >chr8:106506333-106506423 - E=1e-42
CTACATATTTGGAAGTCACTGACCTAGTGACACCTCAGATATGGGTG
```
